# Loss of Two-Pore Channel 2 (TPC2) Expression Increases the Metastatic Traits of Melanoma Cells by a Mechanism Involving the Hippo Signalling Pathway and Store-Operated Calcium Entry

**DOI:** 10.3390/cancers12092391

**Published:** 2020-08-24

**Authors:** Antonella D’Amore, Ali Ahmed Hanbashi, Silvia Di Agostino, Fioretta Palombi, Andrea Sacconi, Aniruddha Voruganti, Marilena Taggi, Rita Canipari, Giovanni Blandino, John Parrington, Antonio Filippini

**Affiliations:** 1 Department of Anatomy, Histology, Forensic Medicine and Orthopaedics, Unit of Histology and Medical Embryology, SAPIENZA University of Rome, 16 Via A. Scarpa, 00161 Roma, Italy; antonella.damore@uniroma1.it (A.D.); fioretta.palombi@uniroma1.it (F.P.); marilena.taggi@uniroma1.it (M.T.); rita.canipari@uniroma1.it (R.C.); antonio.filippini@uniroma1.it (A.F.); 2 Department of Pharmacology, University of Oxford, Mansfield Road, Oxford OX1 3QT, UK; ali.hanbashi@worc.ox.ac.uk (A.A.H.); aniruddha.voruganti@exeter.ox.ac.uk (A.V.); 3 Department of Pharmacology, College of Pharmacy, Jazan University, Jazan 45142, Saudi Arabia; 4 Oncogenomic and Epigenetic Unit, Istituto di Ricovero e Cura a Carattere Scientifico (IRCCS), Regina Elena National Cancer Institute, 00144 Rome, Italy; silvia.diagostino@ifo.gov.it (S.D.A.); giovanni.blandino@ifo.gov.it (G.B.); 5 Biostatistica e Bioinformatica, Clinical Trial Center, Istituto di Ricovero e Cura a Carattere Scientifico (IRCCS), Regina Elena National Cancer Institute-IFO, 00144 Rome, Italy; sacconiandrea@hotmail.com

**Keywords:** TPC2, HIPPO, melanoma, SOCE, metastasis

## Abstract

Melanoma is one of the most aggressive and treatment-resistant human cancers. The two-pore channel 2 (TPC2) is located on late endosomes, lysosomes and melanosomes. Here, we characterized how TPC2 knockout (KO) affected human melanoma cells derived from a metastatic site. TPC2 KO increased these cells’ ability to invade the extracelullar matrix and was associated with the increased expression of mesenchymal markers ZEB-1, Vimentin and N-Cadherin, and the enhanced secretion of MMP9. TPC2 KO also activated genes regulated by YAP/TAZ, which are key regulators of tumourigenesis and metastasis. Expression levels of ORAI1, a component of store-operated Ca^2+^ entry (SOCE), and PKC-βII, part of the HIPPO pathway that negatively regulates YAP/TAZ activity, were reduced by TPC2 KO and RNA interference knockdown. We propose a cellular mechanism mediated by ORAI1/Ca^2+^/PKC-βII to explain these findings. Highlighting their potential clinical significance, patients with metastatic tumours showed a reduction in TPC2 expression. Our research indicates a novel role of TPC2 in melanoma. While TPC2 loss may not activate YAP/TAZ target genes in primary melanoma, in metastatic melanoma it could activate such genes and increase cancer aggressiveness. These findings aid the understanding of tumourigenesis mechanisms and could provide new diagnostic and treatment strategies for skin cancer and other metastatic cancers.

## 1. Introduction

Melanoma originates from the tumoral transformation of melanocytes in the skin [1]. The depth of the invasion and the metastasis of the lymph nodes are very important prognostic features for the classification of melanoma; typically, this type of cancer is associated with a very heterogeneous tumour characterized by high mutational burden (e.g., *BRAF*, *NRAS*, *PTEN*, *TP53*, *CDKN2A* are the most frequently muted genes) and because of this, it is considered a particularly aggressive type of cancer [2]. For this reason, a greater understanding of the molecular and cellular mechanisms underlying melanoma tumourigenesis and metastasis and the identification of new therapeutic targets are both important goals for cancer research. Two-pore channels (TPCs) are members of the voltage-gated cation channel superfamily [3]. Only two isoforms are present in humans and other primates: TPC1 and TPC2. TPC2 is present in the membranes of lysosomes, late endosomes and also melanosomes. There is currently a debate about the precise mechanism of action and mode of regulation of TPC2, both in terms of the protein’s biophysical characteristics, and whether it is primarily activated by nicotinic acid adenine dinucleotide phosphate (NAADP), or by phosphatidylinositol 3,5-bisphosphate (PI(3,5)P_2_) [4,5].

Thus contrasting studies have indicated that TPCs are highly selective for Na^+^ and are regulated by [PI(3,5)P_2_] but not NAADP [4,5], or alternatively that TPCs mediate Ca^2+^ release evoked by NAADP [6,7,8,9]. Possible explanations for such contrasting data are that NAADP may not directly interact with TPCs, but through an accessory binding protein [10,11], and that Mg^2+^ can affect NAADP-evoked TPC2 Na^+^ currents [12]; thus, different experimental conditions may influence the mode of TPC activation. In line with such a possibility, a recent study demonstrated the different effect of two novel TPC2 agonists; one of which induced Ca^2+^ release and the other Na^+^ release. These agonists appear to mimic the actions of NAADP and PI(3,5)P_2_, suggesting that the two physiological agonists bind different sites on the TPC2 ion channel and regulate different processes mediated by this protein [13].

We have previously demonstrated the involvement of TPC2 in one potential aspect of tumourigenesis, namely neo-angiogenesis. By using TPC1 and TPC2 knock-out in vivo models, we reported that VEGF-induced neo-angiogenesis occurred through the regulation of the VEGFR2/NAADP/TPC2/Ca^2+^ signalling pathway [14]. More generally, Ca^2+^ signals have been shown to play a role in cancer, including melanoma, in various ways [15]. Store-operated calcium entry (SOCE) is an important Ca^2+^ signalling mechanism. The depletion of Ca^2+^ from the endoplasmic reticulum (ER) induces plasma membrane Ca^2+^ influx [16]. SOCE is mediated by the endoplasmic Ca^2+^ depletion sensor (STIM1), which translocates to the plasma membrane and activates the Ca^2+^ influx channel (ORAI1). Previous studies have shown that the role of SOCE may be different between metastatic melanoma cells derived from patients, and those without metastatic activities [17]. Intriguingly, a known link exists between endolysosomal Ca^2+^ and SOCE, since it has also been shown that Ca^2+^ release from the endolysosomal compartment can trigger SOCE in primary cultured neurons [18]. Moreover, TFEB has been identified as a novel regulator of intracellular Ca^2+^ homeostasis, with the lysosomal control of SOCE being proposed as an important aspect of lysosomal function and cellular health [19]. TFEB-dependent reduction of SOCE is dependent on lysosomal dynamics. TFEB knockdown reduced the number of lysosomes and their localization to the plasma membrane.

In recent years, the HIPPO pathway has been demonstrated to modulate cell proliferation and differentiation, and to contribute to the progression of a number of diseases, including cancer [20]. Mammalian Ste20-like kinases 1/2 (MST1/2) and the large tumour suppressor 1/2 (LATS1/2) are components of the kinase cascade of the HIPPO pathway [21]. Following the activation of the HIPPO pathway, MST1/2 is phosphorylated and activates LATS1/2, which can then phosphorylate the yes association protein (YAP) and its paralogue, the transcriptional coactivator with PDZ-binding motif (TAZ), resulting in the inhibition of the transcriptional activity of YAP/TAZ [20]. This phosphorylation promotes YAP/TAZ cytoplasmic localisation; when not inhibited in this way, the translocation of YAP/TAZ to the nucleus promotes the activation of their target genes which play key roles in cell growth, proliferation and organ development [22]. Interestingly, the hyperactivation of YAP has been shown to be associated with poor cancer prognosis [23].

Here, we studied whether TPC2 may play different roles in melanoma in terms of primary and metastatic tumours, and also demonstrate for the first time a connection between TPC2, Ca^2+^ signalling, and the activation of the HIPPO signalling pathway.

## 2. Results

### 2.1. Association between TPC2 Expression and the Prognosis of Melanoma Patients

**Figure 1:**
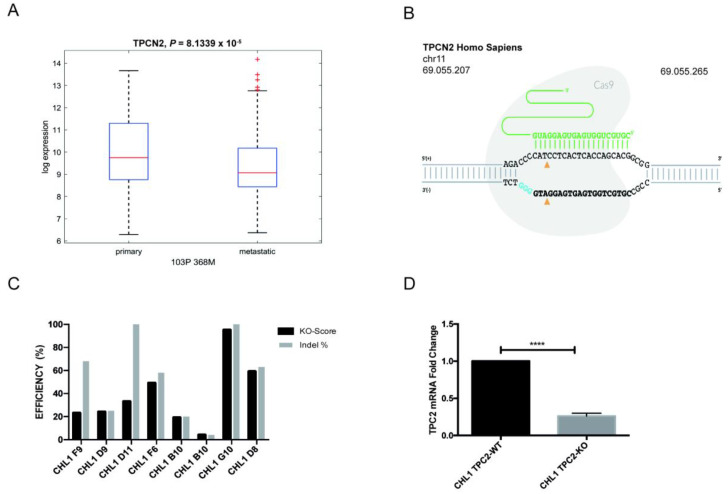
TPC2 expression in the melanoma patient dataset and the generation of a human melanoma cell model TPC2 knockout. (**A**) Analysis of expression and prognostic value for TPC2 between primary and metastatic patients in human skin cutaneous melanoma (SKCM). Dashed line indicates 95% confidence interval (*p*-value: 8.1339 × 10^−5^). (**B**) Schematic of CRISPR-Cas9 strategy used to target CHL1 human melanoma cell line. (**C**) Interference of CRISPR Edits (ICE) Analysis of possible knockout (KO) clones, from which the CHL1 clone G10 was selected. KO score indicates the percentage of sequences that are putative knockouts. (**D**) qPCR analysis of TPC2 transcript levels in CHL1 (wild type (WT) and KO). Data in bar charts represent the mean ± s.e.m. of three independent experiments. **** *p* < 0.0001.

In recent years, with the development of high-throughput RNA sequencing (RNA-Seq), large amounts of RNA-Seq data have emerged. To evaluate the prognostic role of TPC2 (gene name *TPCN2*) in skin cutaneous melanoma (SKCM), we analysed *TPCN2* mRNA expression levels in the Cancer Genome Atlas (TCGA). We identified a significant difference in *TPCN2* mRNA expression between primary and metastatic patients. The reduction of TPC2 expression in the metastatic patients could indicate a different prognostic role for TPC2 in these two stages of cancer (Figure 1A).

### 2.2. TPC2 Depletion Impairs Human Melanoma Cell Adhesion Mediated by α2β1 Integrin Receptor

Amelanotic melanoma is a rare form of human melanoma cancer and generally difficult to diagnose in the first stage due to its lack of pigmentation. Therefore, finding new markers to recognize this form of melanoma at an early stage of tumourigenesis is an important goal that could have major therapeutic benefits. CHL1 cells are a model of a human amelanotic melanoma and are derived from a metastatic site.

We first used CRISPR/Cas9 genome editing to knockout (KO) the *TPCN2* gene in CHL1 and B16-F0 (murine primary melanoma) cell lines. Three different single guide RNAs (sgRNAs) were used to target *TPCN2* exon 4 with the aim of generating a frame shift in the TPC2 open reading frame (ORF) via non-homologous end-joining (NHEJ), in each cell line (Figure 1B and Figure S1A); this targeting was followed by CRISPR editing analysis using an Interference of CRISPR Edits (ICE) tool to select clones with the highest KO score (Figure 1C and Figure S1B). Furthermore, *TPCN2* mRNA expression in each putative TPC2 KO cell line was assessed (Figure 1D and Figure S1C).

**Figure 2:**
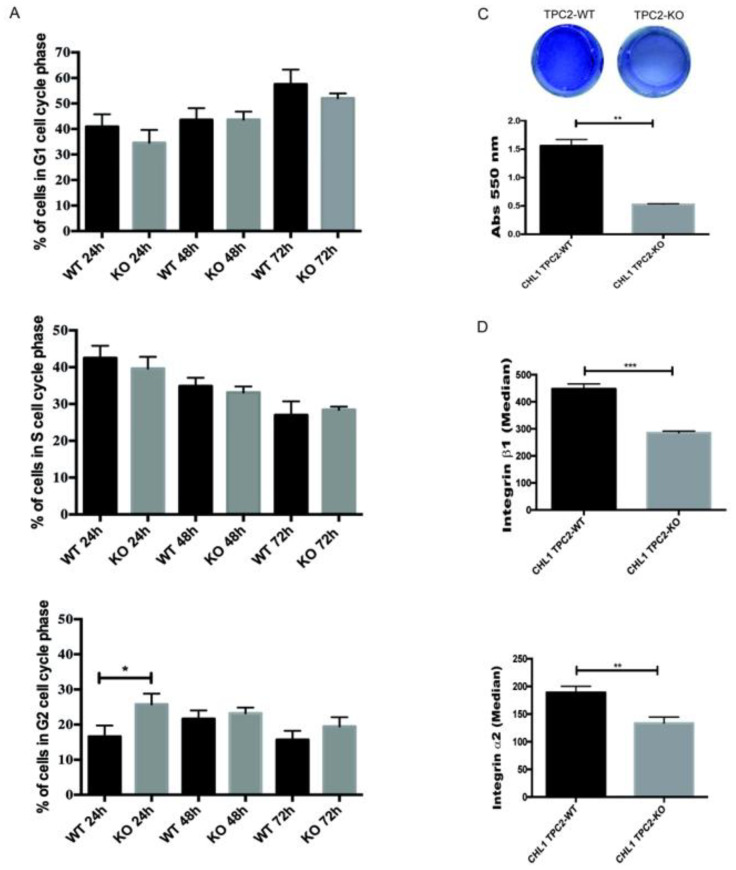

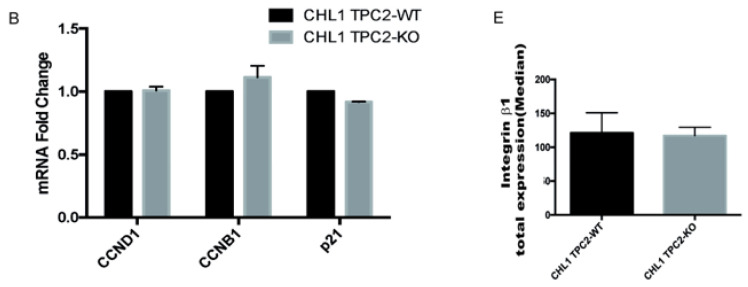
TPC2 KO impairs cell adhesion to collagen type I matrix. (**A**) Cell cycle analysis at 24, 48, and 72 h of CHL1 WT and TPC2 KO cells. (**B**) qPCR analysis of cell cycle markers CCND1, CCNB1 and p21; H3 was used as an internal control. (**C**) Adhesion assay on collagen type I matrix, cells stained with crystal violet. (**D**) Flow cytometric detection of α2β1 integrin chain in CHL1 WT and TPC2 KO cells. (**E**) Flow cytometric detection of Integrin-β1 on CHL1 WT and TPC2 KO fixed and permeabilized cells. Data in bar charts represent the mean ± s.e.m. of three independent experiments (* *p* < 0.05; ** *p* < 0.01; *** *p* < 0.001).

Then, we investigated the proliferation capacity of CHL1/TPC2 KO cells. TPC2 KO cells showed a slower G2 phase compared to wild type (WT) cells, after 24 h from seeding (Figure 2A). After 48 h from seeding, the TPC2 KO cell line recovered the proliferation rate of the control cell line (Figure 2A). These data are further corroborated by the analysis of the cell cycle markers cyclin D1 (CCND1) for the G1/S phase, cyclin B1 (CCNB1) for the G2/M phase, and of the cyclin-dependent kinase inhibitor 1 (p21) as a marker of cell cycle arrest. These transcripts showed no significant difference in expression (Figure 2B).

Subsequently, we performed an adhesion assay by using cell plates coated with collagen type I matrix to assess metastatic traits after TPC2 KO. Interestingly, TPC2 KO cells showed a drastically reduced ability to bind this matrix (Figure 2C). Looking further, we investigated the expression of α2 and β1 integrin chains that together form the main receptor to collagen type I. Plasma membrane expression of both these adhesion proteins in live cells showed a significant reduction in the TPC2 KO cells (Figure 2D). In contrast, the whole cell expression of β1-integrin did not change (Figure 2E). These findings show similarity to those of Nguyen et al. [24] who showed that TPC2 down regulation in bladder carcinoma cells is related to a halted trafficking of β1-integrins. These combined findings strongly suggest that TPC2 is involved in tumour metastasis rather than in cancer cell proliferation.

### 2.3. TPC2 Inhibition in Human Melanoma Cells Increases Their Invasion Capability

**Figure 3:**
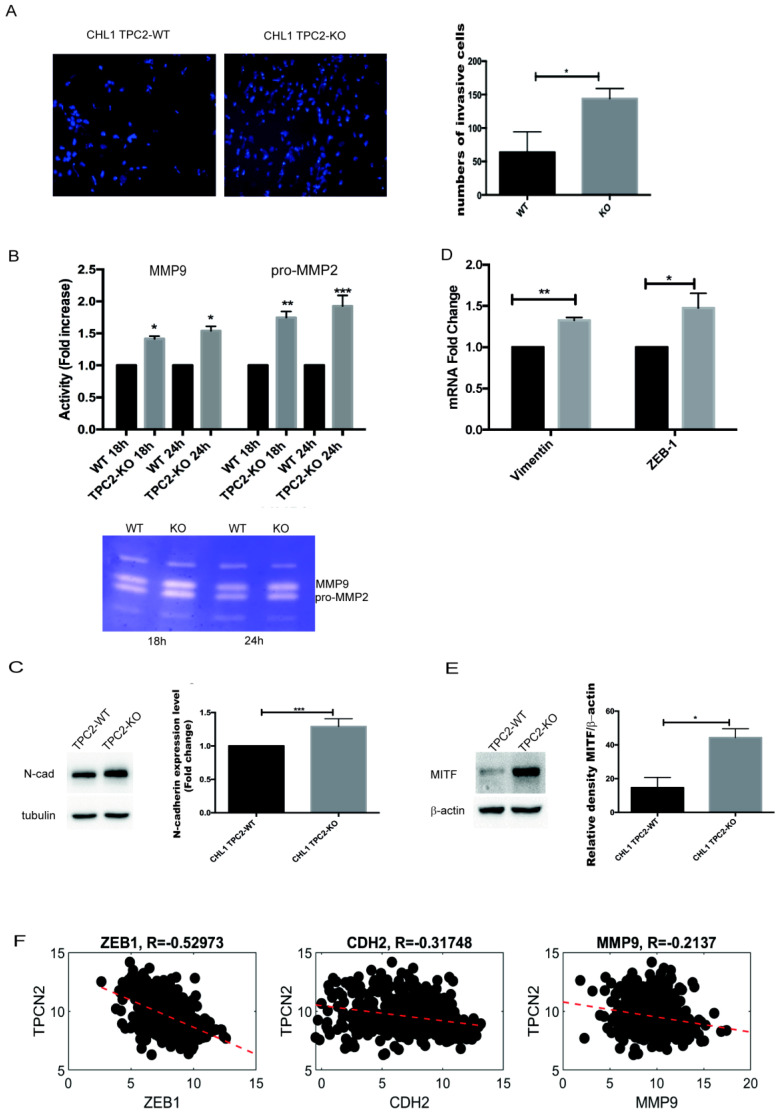
TPC2 depletion increases metastatic melanoma cell invasion. (**A**) Transwell assay after 24 h incubation on matrigel matrix, cell nuclei stained with DAPI. (**B**) Proteolytic Matrix Metalloproteinases (MMPs) activity in CHL1 WT and TPC2 KO cells detected by zymography. Active MMP-9 (82 kDa) and proMMP-2 (72 kDa). (**C**) N-cadherin protein expression in CHL1 WT and TPC2 KO cells, normalised to tubulin. Relative density of N-cadherin/tubulin. (**D**) qPCR analysis of vimentin and ZEB1 in CHL1 WT and TPC2 KO cell lines. H3 was used as an internal control. (**E**) MITF protein expression in CHL1 WT and TPC2 KO normalised to β-actin. Relative density of MITF/β-actin. (**F**) Correlation between TPCN2 and ZEB1, N-Cadherin (CDH2), and MMP-9 in human SKCM. Data in bar charts represent the mean ± s.e.m. of three independent experiments (* *p* < 0.05; ** *p* < 0.001; *** *p* < 0.001).

To further explore the role of TPC2 in metastasis, we compared WT and TPC2 KO CHL1 cells’ ability to invade a matrigel substrate. After 24 h, TPC2 KO cells were more invasive (Figure 3A). In line with this, the TPC2 KO cells showed an increased secretion of matrix metalloproteinase 9 (MMP9), as highlighted by zymography assays (Figure 3B).

The epithelial–mesenchymal transition (EMT) is an evolutionarily conserved developmental process that confers metastatic properties upon cancer cells by enhancing their mobility, invasion, and resistance to apoptotic stimuli. A common feature of EMT is the loss of epithelial cadherin (E-cadherin) expression and the concomitant up regulation or de novo expression of neural cadherin (N-cadherin). Analysing E- and N-cadherin expression, we found that neither WT nor TPC2 KO cells expressed E-cadherin but in TPC2 KO cells the expression of N-cadherin was increased (Figure 3C). We also analysed the expression of the transcription factor zinc-finger E-box-binding homeobox1 (ZEB-1), a known inducer of EMT [25], and vimentin, the major component of the intermediate filaments, which is normally expressed in mesenchymal cells [26], and considered a mesenchymal marker. ZEB-1 and vimentin were up regulated in TPC2 KO cells (Figure 3D). All these data suggest that TPC2 KO cells are more mesenchymal and so more invasive. Melanocyte Inducing Transcription Factor (MITF) expression promotes melanoma cell survival and migration [27,28]. TPC2 KO cells showed a higher level of expression of the MITF protein (Figure 3E).

Moreover, we analysed the clinical correlation of TPC2 expression with that of some of the genes which correlated with TPC2 expression in our in vitro studies. There was a significant negative correlation between TPC2 and ZEB1 (R = −0.535, *p* < 0.05); N-cadherin (CDH2) (R = − 0.317, *p* < 0.05) and MMP9 (R = −0.213, *p* < 0.05) (Figure 3F) in the human SKCM dataset. We also analysed this correlation among primary and metastatic patients (Figures S3 and S4; Table S1).

### 2.4. TPC2 and HIPPO Pathway Connection in CHL1 and MeWo Metastatic Cell Lines

**Figure 4:**
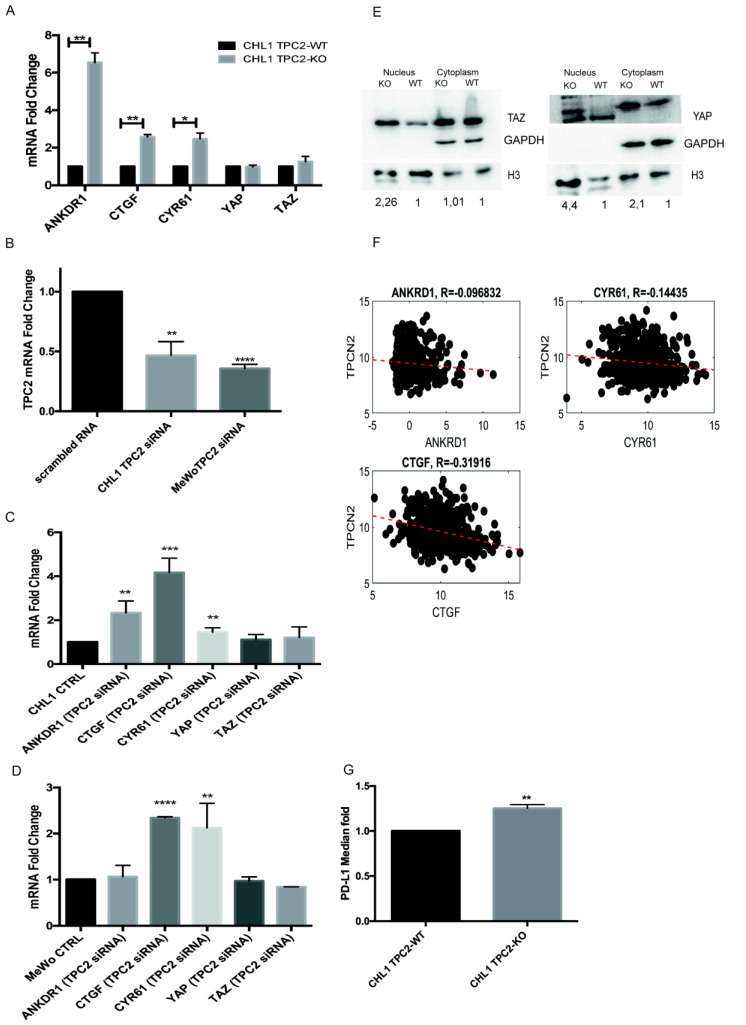
TPC2 KO and silencing induces YAP/TAZ target genes. (**A**) qPCR analysis of YAP/TAZ target genes in CHL1 WT and TPC2 KO cells. H3 was used as an internal control. (**B**) TPC2 transient inhibition after 24 h from CHL1 transfection. (**C**) qPCR analysis of YAP/TAZ target genes after the transient silencing of TPC2 in CHL1 cells. Glyceraldehyde 3-phosphate dehydrogenase (GAPDH) was used as an internal control. (**D**) qPCR analysis of YAP/TAZ target genes after the transient silencing of TPC2 in the MeWo cell line. GAPDH was used as an internal control. (**E**) Nucleus–cytoplasm extraction to analyse YAP and TAZ nuclear localization. (**F**) Correlation between TPCN2 and CTGF, CYR61, and ANKRD1 in human SKCM (for the *p*-value see Table S1). (**G**) Flow cytometric detection of PD-L1 in CHL1 WT and TPC2 KO cells after IFN-γ induction. Data in bar charts represent the mean ± s.e.m. of three independent experiments (* *p* < 0.05; ** *p* < 0.01; *** *p* < 0.001; **** *p* < 0.0001).

To gain insight into the molecular mechanisms underlying the apparent increased aggressiveness of CHL1 TPC2 KO cells, we investigated the potential links with an important emerging pathway associated with tumour initiation and progression. The emerging role of YAP/TAZ and the transcriptional effectors of HIPPO signalling, as the key drivers of tumour initiation and growth, are major recent discoveries in cancer research. Their activity can control proliferation, invasion and metastasis and is usually associated with poor prognosis [29]. High YAP levels correlate with decreased survival in melanoma patients [23] and TAZ activation has been linked to lung cancer brain metastasis [30]. To assess the activation status of YAP/TAZ, we analysed the expression of ankyrin repeat domain-containing protein (ANKRD1), cysteine-rich 61 (CYR61), and connective tissue growth factor (CTGF), which are considered to be the bona fide YAP/TAZ target genes [31]. Notably, all these target genes were strongly up regulated in TPC2 KO cells, indicating YAP/TAZ transcriptional activation (Figure 4A).

In order to independently study whether there is a correlation between a reduction of TPC2 expression and the enhanced expression of YAP/TAZ target genes, we used RNA interference (RNAi) to transiently silence TPC2 expression for 24 h in the CHL1 and MeWo metastatic melanoma cell lines (Figure 4B). Importantly, using this completely different approach to supress TPC2 expression, we observed increased expression levels of ANKRD1 (but only for CHL1, not for MeWo), CTGF, and CYR61 mRNAs, in TPC2-silenced cells (Figure 4C,D). We also studied whether the activation of YAP/TAZ target genes that occurs in TPC2 KO cells is associated with the increased movement of YAP/TAZ from the cytoplasm to the nucleus. By differential nucleus-cytoplasm protein extraction, we confirmed an increase in YAP and TAZ in the nucleus in the TPC2 KO cells (Figure 4E).

Collectively, these data confirm a link between the loss of, or reduction of, TPC2 expression and the transcriptional activation of YAP/TAZ target genes linked to the movement of these transcriptional activators from the cytoplasm to the nucleus. These results shed a light on a novel role for TPC2 at a late stage of cancer.

To further validate these findings in melanoma patients, we studied the clinical correlation of TPC2 expression with that of YAP/TAZ target genes, in the skin melanoma TCGA database. There was a significant negative correlation between TPC2 and CTGF (R = −0.311, *p* < 0.05); CYR61 (R = −0.134, *p* < 0.05), but not with ANKDR1 (R = −0.0932, *p* = 0.07) (Figure 4F). We also analysed this correlation among primary and metastatic patients (Figures S3 and S4; Table S1).

### 2.5. PD-L1 Induced by INTERFERON-γ Is Significantly Higher in CHL1 TPC2 KO Cells

It has previously been demonstrated that TAZ can regulate the transcription of PD-L1 [32]. PD-L1 is one of the ligands of PD-1, which represents a target for advanced melanoma immunotherapy [33]. PD-L1 shows abnormally high expression in tumour cells and is considered the main factor responsible for promoting the ability of tumour immune escape [34]. After treatment with 100 ng/mL Interferon-γ (IFN-γ), CHL1 TPC2 KO cells displayed an increased level of surface expression of PD-L1 (Figure 4G), providing further indirect confirmation of the activation of the YAP/TAZ pathway after TPC2 KO.

### 2.6. TPC2 Regulates YAP/TAZ Activity Modulating ORAI 1 Channel Expression

**Figure 5:**
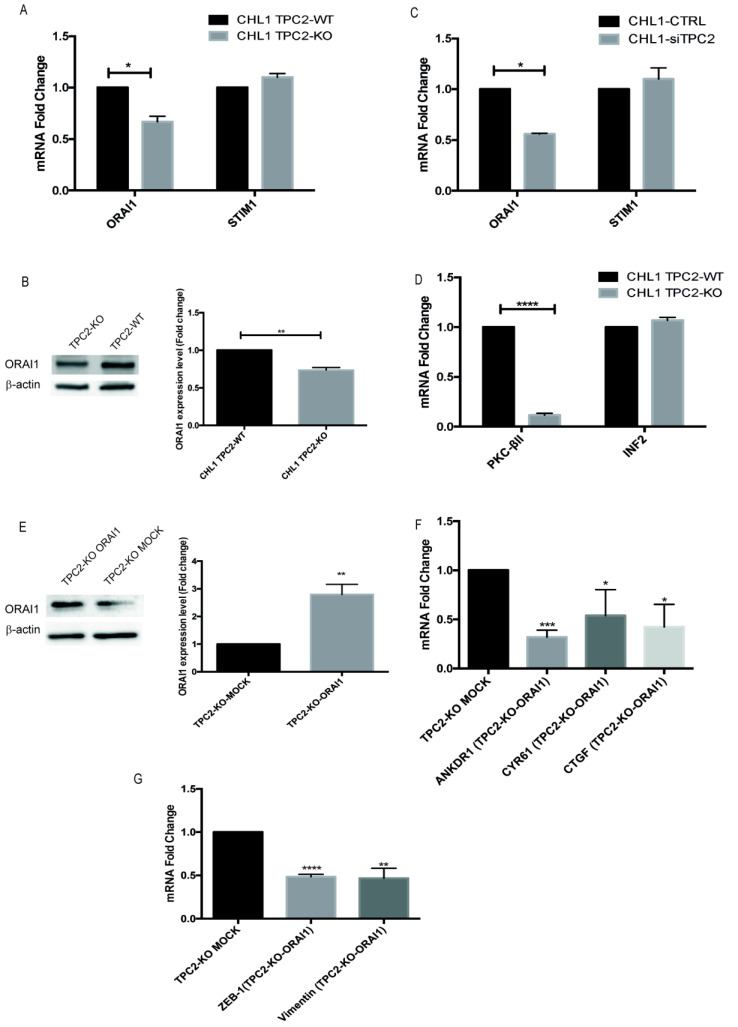
Possible mechanism involved in YAP/TAZ regulation mediated by TPC2. (**A**) qPCR analysis of ORAI1 and STIM1 in CHL1 WT and TPC2 KO cells. (**B**) ORAI1 protein expression in CHL1 WT and TPC2 KO cells normalised to β-actin. Relative density of ORAI1/β-actin. (**C**) qPCR analysis of ORAI1 and STIM1 expression after the transient silencing of TPC2. (**D**) qPCR analysis of PKC-βII and INF2 expression in CHL1 WT and TPC2 KO cells. (**E**) ORAI1 protein expression after 24 h from transfection in CHL1 TPC2 KO-MOCK and ORAI1 expressing cells, normalised to β-actin. Relative density of ORAI1/β-actin. (**F**) qPCR analysis of YAP/TAZ target gene expression after ORAI1 overexpression in CHL1 TPC2 KO cells. H3 was used as an internal control. (**G**) qPCR analysis of vimentin and ZEB1 expression after ORAI1 overexpression in CHL1 TPC2 KO cells. H3 was used as an internal control. Data in bar charts represent the mean ± s.e.m. of three independent experiments (* *p* < 0.05; ** *p* < 0.01; *** *p* < 0.001; **** *p* < 0.0001).

An important question is how loss of expression of an endolysosomal ion channel like TPC2 affects YAP/TAZ activity. Previously, we and others have shown that TPC2 is a mediator of Ca^2+^ signalling responses in cells [7,14]. To date, the only known link between YAP/TAZ activity and Ca^2+^ signalling is via store-operated Ca^2+^ entry (SOCE), a process that replenishes the endoplasmic reticulum (ER) Ca^2+^ store through an interaction between the ER protein STIM1 and the plasma membrane ion channel ORAI1 [35]. A study in glioblastoma cells showed that the stimulation of SOCE inhibited YAP/TAZ activity via the activation of PKC-βII [36]. This kinase regulates MST1/2 and LATS 1/2 phosphorylation, both signalling components in the HIPPO pathway. When they are activated by phosphorylation, YAP/TAZ are phosphorylated and sequestered in the cytosol. The glioblastoma study also showed that the elevated level of Ca^2+^ induced by SOCE led to actin cytoskeleton remodelling mediated by INF2 [36]. In the current study we compared the expression levels of ORAI1 and PKC-βII. Interestingly, TPC2 KO cells showed a substantial down regulation of both ORAI1 and PKC-βII expression (Figure 5A–C); in contrast, STIM1 and INF2 expression were unchanged. We also observed that ORAI1 expression is reduced after TPC2 transient silencing (Figure 5D). To confirm the connection between ORAI1, TPC2 and YAP/TAZ, we performed a rescue of ORAI1 protein in our TPC2 KO model, and we saw a reduction of YAP/TAZ target gene expression compared to the mock-transfected cells when ORAI1 is overexpressed (Figure 5E,F). We also analysed vimentin and ZEB1 levels and showed that they are decreased when ORAI1 is overexpressed (Figure 5G). To study whether mechanotransduction may play a role in YAP/TAZ activation following TPC2 KO, we investigated the activation of YAP/TAZ target genes and N-Cadherin in ultra-low cell attachment conditions and found no such activation in these circumstances (Figure S2). These findings suggest that the loss of TPC2 may lead to the activation of YAP/TAZ target genes, and therefore increased metastasis, because of changes in the capacity of both SOCE and the cytoskeleton to mediate the inhibition of the HIPPO pathway.

### 2.7. TPC2 Is Overexpressed while YAP/TAZ Target Genes Are Down Regulated after Vemurafenib Treatment in A375 Melanoma Cells

**Figure 6:**
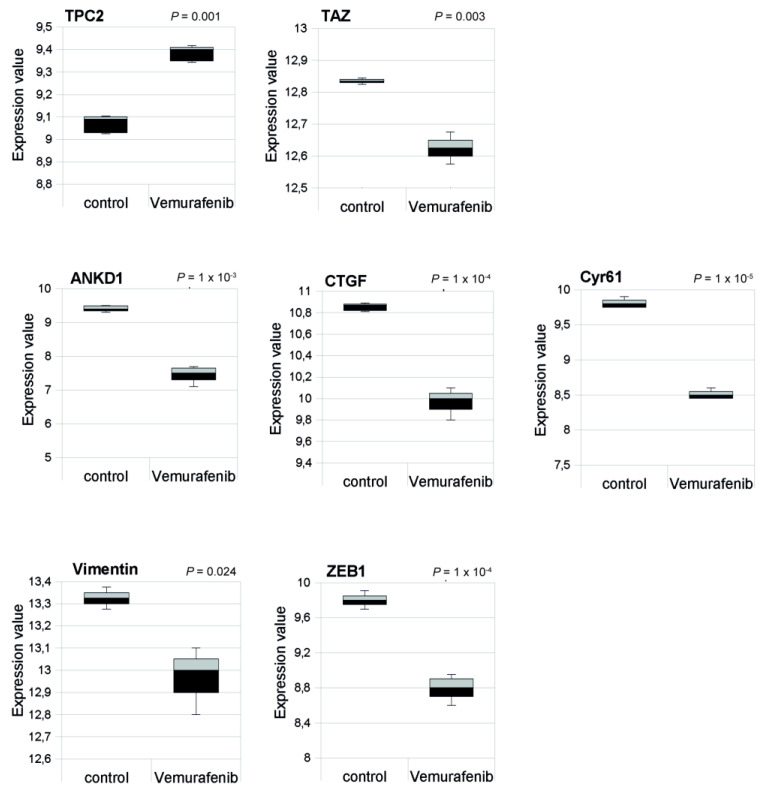
Analysis of A375 melanoma cells harbouring the BRAF V600E oncogenic mutation following treatment with the BRAF inhibitor Vemurafenib.

To further support the link between the reduction of TPC2 expression and YAP/TAZ target gene activation, we also analysed a dataset from another study [37] that investigated the effect of the BRAF inhibitor Vemurafenib on the BRAFV600E A375 melanoma cell line, the mutant form of BRAF in this cell line being differentially sensitive to inhibition by Vemurafenib (Figure 6). Studying this dataset, we noticed that after Vemurafenib treatment, TAZ and its target genes CTGF, CYR61, and ANKRD1 were down regulated, while TPC2 was overexpressed. Moreover, vimentin and ZEB1 were down regulated after Vemurafenib treatment, indicating a reduction of mesenchymal phenotype [37]. By indicating that YAP/TAZ activity is decreased when TPC2 expression is increased, such findings from a completely independent study are in line with our findings that indicate an inverse relationship between TPC2 expression and YAP/TAZ activity.

## 3. Discussion

TPC2 has been previously demonstrated to play a role in cancer progression. In particular, Nguyen et al. [24] have shown that silencing and pharmacologically inhibiting this channel impaired the migration of urinary bladder and hepatic human carcinoma cells in vitro, and of murine breast cancer cells both in vitro and in an in vivo mouse model. In contrast, our current study identifies for the first time a role for TPC2 in the aggressiveness of metastatic melanoma, and strongly indicates that in human metastatic melanoma cells, this role appears to differ from what is currently known from studying human cancer cells derived from primary tumours.

At the outset of this study, we performed a bioinformatic analysis to evaluate the potential prognostic role of TPC2 in human SKCM. Interestingly, we identified a significant difference in the expression of TPC2 between primary and metastatic patients, with TPC2 expression being significantly reduced in the latter. This suggests that the role played by TPC2 in primary and metastatic tumours might be qualitatively different.

To study the role of TPC2 in melanoma tumourigenesis and metastasis, we generated a CHL1 TPC2 KO cell line, CHL1 cells being a model of human amelanotic melanoma and derived from a metastatic site. They expressed a wild-type BRAF kinase and a mutant TP53 protein. Given these premises, CHL1 is considered a metastatic melanoma cell line. In this model, we observed that the loss of TPC2 expression reduced the ability of these cells to bind the collagen type I matrix. This binding is mediated by the α2β1 integrin receptor, which was also reduced in the TPC2 KO cells. Moreover, the ability of CHL1 cells to invade a matrigel matrix was also enhanced by TPC2 KO, and this was associated with an increased activity of MMP9, a protein involved in cell invasion. Combined, these findings strongly indicate that TPC2 KO cells are more invasive than their WT counterparts, a highly unexpected finding given the existing data on TPC2 in different cancer cell models [24].

The analysis of markers of the EMT, a key process in the onset of metastasis, also supports this hypothesis. Thus, the TPC2 KO cells showed increased expression levels of ZEB-1, N-cadherin and vimentin, indicating that TPC2 KO cells are more mesenchymal. Furthermore, MITF, which is amplified in up to 20% of melanomas, with higher incidence among metastatic melanoma samples [38], was more highly expressed in TPC2 KO cells. Interestingly, a significant negative correlation between TPC2 and ZEB1, N-cadherin, and MMP9 was also found in the human SKCM dataset.

Significantly, some of the YAP/TAZ target genes, such as ANKRD1, CYR61, and CTGF, were also strongly up regulated in the TPC2 KO cells. YAP/TAZ are drivers of tumour initiation and growth. Their activity can control proliferation, invasion and metastasis, and is usually associated with poor prognosis [29]. It has also been shown that high levels of YAP are correlated with a decrease in survival for melanoma patients [23].

Studies of the effect of gene knockout on the properties of cells in culture can benefit from using parallel, independent approaches to achieving a reduction of a gene’s expression. Importantly, we also confirmed that the transient inhibition of TPC2 in the CHL1 and MeWo metastatic human cell lines via RNAi using anti-TPC2 siRNAs, has similar effects on the expression of YAP/TAZ target genes. We also showed the translocation of YAP and TAZ from the cytoplasm to the nucleus in TPC2 KO cells, indicating the activation of this pathway. Moreover, this activation did not occur in ultra-low cell attachment conditions, suggesting a link with mechanotransduction in the activation of these factors that is associated with the reduction in TPC2 expression. Thus, these data suggest an aggressive phenotype for TPC2 KO cells. Furthermore, looking to the potential clinical significance of these findings by the use of the skin melanoma TCGA database, we also found a significant negative correlation between TPC2 expression and that of the YAP/TAZ target genes CTGF and CYR61, although intriguingly, there was no significant correlation with another target gene, ANKRD1. Interestingly, we also found a lack of ANKRD1 expression induction in MeWo TPC2-silenced cells, maybe indicating a differential effect of reduced TPC2 expression on this gene in distinct types of metastatic cancer cells.

Further studies will be required in the future to explore why there may be such a differential effect of TPC2 KO on the expression of CTGF, CYR61, and ANKRD1 in some circumstances, which could be revealing in terms of its implications for our understanding of the mechanisms of activation of these genes by YAP/TAZ.

Further indications of the increased aggressiveness of TPC2 KO cells comes from our findings relating to the induction of the expression of PD-L1. This suggests the increased ability of these cells to escape the immune system [34].

The transcription of PD-L1 has been shown to be regulated by TAZ [32], but this relationship between TAZ and PD-L1 is not conserved in a number of mouse cell lines, likely due to differences between the human and mouse PD-L1 gene promoters [32]. In the murine model of melanoma B16-F0 cells, the amount of PD-L1 exposed on the membrane of TPC2 KO cells was reduced (Figure S1D). Furthermore, in this model we did not see any YAP/TAZ pathway activation (Figure S1E). These data indicate a difference between the human and murine melanoma models studied but could also support a different role for TPC2 in melanoma according to the stage of the pathology as B16-F0 cells are derived from a primary tumour. One could speculate that the breast cancer murine line 4T1 in Nguyen et al. [24] would also not be associated with the activation of the YAP/TAZ pathway, due to the different regulation of this pathway between murine and human species. Further studies of animal and human WT and TPC2 KO melanoma cell lines derived from tumours at different stages of metastatic progression will be required to address this issue more definitively. However, our analysis of an independent study by Parmenter et al. [37] showed that after Vemurafenib treatment, TPC2 is overexpressed, while YAP/TAZ target genes are down regulated as well as ZEB1 and Vimentin, confirming our data in a different model of melanoma.

A key outstanding question is how a reduction in the expression of an endolysosomal ion channel would manifest itself in an increase in the metastatic traits of melanoma cells mediated by YAP/TAZ. Possibly, this occurs through a change in endolysosomal pH. Changing TPC2 expression could affect lysosomal or melanosomal pH either by increasing melanosomal and lysosomal pH when TPC2 is genetically inhibited [39,40,41] or decreasing lysosomal pH if TPC2 is overexpressed or pharmacologically activated [13,42]. However, it remains to be shown how such a change in endolysosomal pH would affect the HIPPO pathway, which is the primary mediator of YAP/TAZ activity.

Given previous evidence that TPC2 is a mediator of Ca^2+^ signalling responses, one obvious possibility is that changes in such responses underlie how a loss of, or a reduction in, TPC2 expression levels are associated with an increase in YAP/TAZ activity, and as a consequence, an enhancement of metastatic traits. However, the only published link between Ca^2+^ signalling responses and TAP/TAZ activity has implicated SOCE, a process that replenishes the endoplasmic reticulum (ER) Ca^2+^ store through an interaction between the ER protein STIM1 and the plasma membrane ion channel ORAI1 [35]. Thus, a study in glioblastoma cells has shown that the stimulation of SOCE inhibited YAP/TAZ activity via the activation of PKC-βII [36].

To see whether changes in SOCE might underlie the link between changes in TPC2 expression and YAP/TAZ activity, we analysed the expression of ORAI1 and PKC-βII; when PKC-βII is active, this kinase phosphorylates MST1/2 and LATS 1/2, which subsequently phosphorylate YAP/TAZ to remain in the cytosol. We found that in TPC2 KO or anti-TPC2 siRNA-treated cells, both ORAI1 and PKC-βII levels decreased. This suggests that an inhibition of SOCE associated with a loss of, or reduction of, TPC2 expression, may allow YAP/TAZ to translocate into the nucleus and activate their target genes. Indeed, when ORAI1 was overexpressed in TPC2 KO cells, YAP/TAZ target genes were down regulated. This raises the question of how changes in the expression of TPC2, an endolysosomal ion channel, could cause such a change in the levels of expression of ORAI1 and PKC-βII. One obvious possibility is that such changes are a consequence of a functional inhibition of SOCE, which would be in line with a previously described connection between SOCE and endolysosomal Ca^2+^ release [18,19].

## 4. Materials and Methods

### 4.1. Cell Lines

Human CHL1 and MeWo (ATCC, Manassas, VA, USA), and mouse B16-F0 (ATCC, Manassas, VA, USA), melanoma cell lines were maintained in Dulbecco’s modified Eagle’s medium (Sigma, St. Louis, MO, USA) supplemented with 2 mmol/L glutamine (Sigma, St. Louis, MO, USA), 10% foetal bovine serum (Gibco by Life Technologies, Carlsbad, CA, USA), and antibiotics (P/S, Sigma, St. Louis, MO, USA).

### 4.2. Western Blot Analysis

Following cell lysis, the proteins were separated on SDS-PAGE gels. Subsequently, the gels were blotted overnight at 4 °C onto nitrocellulose membranes (Amersham, Buckinghamshire, UK). The membranes were incubated with the following primary antibodies overnight at 4 °C: anti-MITF (Santacruz Biotch, Dallas, TX, USA), anti-YAP (Cell Signalling, Danvers, MA, USA); anti-TAZ (Sigma, St. Louis, MO, USA); anti-N-Cadherin (Abcam, Cambridge, UK); and anti-ORAI1 (ProSci Inc^TM^, San Diego, CA, USA). The intensity of Western blot bands was quantified by Image Lab (Biorad, Hercules, CA, USA) and normalised using β-actin and tubulin (Sigma, St. Louis, MO, USA), GAPDH (Millipore, Burlington, MA, USA), and H3 (Abcam, Cambridge, UK).

### 4.3. CRISPR Design

Single guide RNAs (sgRNAs) were designed using the Synthego (Menlo Park, CA, USA) Design Tool to minimize off-target effects. The cells were transfected and analysed using Synthego (Menlo Park, CA, USA) protocols. Genotyping was performed using the following TPC2 primer sequences:

Human:

Forward

GATAGGGCGGTTACCATCATC

Reverse

CTCACCGGGTCAAAGTACAA

Mouse:

Forward

GGTATTACTTCGAACGTCTGCCAACGGT

Reverse

TTCAAAGCGCCAAAAGCTCACTAGCAA

### 4.4. Cell Transfection

1 × 10^5^ cells were seeded in a 12-well plate. hTPC2 siRNA (Qiagen, Hilden, Germany) was used at 10 nM. Empty-plasmid and RFP-ORAI1 plasmid ([43] gifted by Prof. Vincenzo Sorrentino) were used at 500 ng/mL. Cells were transfected using Lipofectamine 2000 (Life Technologies, Carlsbad, CA, USA) following the manufacturer’s protocol.

### 4.5. Adhesion Assay

Twelve-well plates were coated with collagen type I (Sigma, St. Louis, MO, USA) and incubated overnight at 4 °C. The following day, the excess matrix was removed and blocked with 0.1% bovine serum albumin (BSA) in Calcium Magnesium free Dulbecco’s PBS (CMF-DPBS) for 1 h at room temperature. Subsequently, 2 × 10^5^ cells were seeded and incubated for 90 min at 37 °C. Each well was stained by crystal violet which was dissolved using 10% acetic acid. The absorbance was read at 550 nm.

### 4.6. Invasion Assay

We seeded 1 × 10^5^ cells on Transwell (Corning, Corning, NY, USA) that was coated with reduced growth factor Matrigel (BD, Franklin Lakes, NJ, USA) and a chemo-attractant gradient of FBS (1% to 20%) was used to favour cell movement. After 24 h incubation, the cells were fixed and stained with DAPI. For each Transwell, 5 fields were analysed by counting the cell numbers using Image J 2.0.0-rc-67/1.52c (US National Institutes of Health, Bethesda, MD, USA).

### 4.7. Flow Cytometry and Cell Cycle Analysis

1 × 10^5^ cells were incubated for 30 min with anti-Integrinβ1 (Santacruz Biotech, Dallas, TX, USA) or anti-Integrinα2 (BD Horizon, Franklin Lakes, NJ, USA) antibodies, and with the secondary antibody anti-mouse Alexa-fluor 488, for another 30 min. For PDL1 (Biolegend, San Diego, CA, USA), after 24 h IFN-γ treatment, 1 × 10^5^ cells were incubated for 30 min in the same buffer with anti-PDL1 antibody. Dead cells were excluded by Sytox Blue Stain (Life Technologies, Carlsbad, CA, USA) or propidium iodide (Sigma, St. Louis, MO, USA). For cell cycle analysis, the cells were fixed with 70% ethanol, washed three times with PBS and stained for 3 h at room temperature with PBS–propidium iodide, then analysed using a CyAn ADP flow cytometer (Beckman Coulter, Brea, CA, USA) and FCS express 5 (De Novo software, Glendale, CA, USA).

### 4.8. Bioinformatic Analysis

Normalized gene expression of skin cutaneous melanoma patients was obtained from the Broad Institute TCGA Genome Data Analysis Center (2016): TCGA data from Broad GDAC Firehose 2016_01_28 run; Broad Institute of MIT and Harvard Dataset. https://doi.org/10.7908/C11G0KM9. The statistical significance of the differential modulation of *TPCN2* gene between subgroups of patients was inferred by Student’s *t*-test (*p*-value = 8.13 × 10^−5^) and with a nonparametric Wilcoxon Rank-Sum test (*p*-value = 8.58 × 10^−5^). Positive and negative association between the genes was evaluated by Spearman’s rank correlation. High and low expression values of a gene for each subgroup of patients were assessed by positive and negative z-scores, respectively. Analyses were performed using MATLAB R2019b software. We analysed the GSE42872 dataset [37] from the Gene Expression Omnibus database (GEO: https://www.ncbi.nlm.nih.gov/gds). GEO2R, an online analysing tool of GEO DataSets, was utilized to analyse differentially expressed genes between A375 melanoma cells harbouring the BRAF V600E oncogenic mutation, and which had been treated, or not, with the BRAF inhibitor Vemurafenib. A *p*-value of <0.05 was used as the cut off criterion to identify significant differential expression between the two groups.

### 4.9. Quantitative Real-Time PCR

Total RNA was isolated using an RNeasy mini kit (Qiagen Hilden, Germany). First-strand cDNA synthesis was performed using a SuperScript III reagent kit (Invitrogen, Carlsbad, CA, USA). Real-time PCR was then carried out with primers specific for ANKDR1, CTGF, CYR61, YAP, TAZ, TPC2, ORAI1, STIM1, PKC-βII, INF2, ZEB1, VIMENTIN, CCND1, CCNB1, and p21 (Table 1), using a Powerup SYBR green master mix (Applied Biosystems, Waltham, Massachusetts, USA). GAPDH and H3 were used as internal controls. Relative mRNA expression levels were calculated using the ΔΔCT method.

### 4.10. Gelatin Zymography for Matrix Metalloproteinases (MMPs) Detection

The supernatant was collected after 18 and 24 h after cell starvation with 1% FBS. Gelatinolytic activity of conditioned media was assayed as previously described [44], followed by gelatine zymography as previously described [14]. The values were normalized to protein content.

### 4.11. Statistical Analysis

Data are presented as the mean ± s.e.m. of results from at least three independent experiments. Student’s *t*-test was used for statistical comparison between the means where applicable (two groups) or ordinary one-way ANOVA (for groups of three or more) * *p* < 0.05; ** *p* < 0.01; *** *p* < 0.001; **** *p* < 0.0001.

## 5. Conclusions

In summary, our data indicate that a loss of, or reduction of, TPC2 expression in a metastatic model of human melanoma increased the aggressiveness of melanoma cells. In particular, we identified a previously undisclosed connection between TPC2 and the YAP/TAZ pathway, which could be an emerging focus for cancer research. One must keep in mind that the interaction of cells with the extracellular surroundings can influence the activation of YAP/TAZ, since the latter are also considered to be mechanosensors and mechanotransducers. Indeed, in line with this, we found no activation of YAP/TAZ target genes in TPC2 KO cells in ultra-low cell attachment conditions. It will therefore be interesting in future studies to investigate the possible role of TPC2 in the modulation of cancer cell mechanotransduction and its different role in primary or metastatic tumours. Together with the findings discussed here, such studies will be important in further characterising the mechanistic link between the endolysomal ion channel TPC2 and metastatic cancer, as well as identifying new ways to diagnose and treat this type of cancer.

## Figures and Tables

**Table 1:** Primer sequences.

Gene	Forward	Reverse
CTGF	AGGAGTGGGTGTGTGACGA	CCAGGCAGTTGGCTCTAATC
CYR61	CCTTGTGGACAGCCAGTGTA	ACTTGGGCCGGTATTTCTTC
ANKRD1	AGTAGAGGAACTGGTCACTGG	TGGGCTAGAAGTGTCTTCAGAT
YAP	GCACCTCTGTGTTTTAAGGGTCT	CAACTTTTGCCCTCCTCCAA
TAZ(WWTR1)	GGCTGGGAGATGACCTTCAC	CTGAGTGGGGTGGTTCTGCT
GAPDH	GGAGCGAGATCCCTCCAAAAT	GGCTGTTGTCATACTTCTCATGG
STIM	ATCTCACAGCTCATGGTATGCTCC	GGAAGGTGCCAAAGAGTGTGTTTC
ORAI	TACTTGAGCCGCGCCAAGCTTAAA	ACCGAGTTGAGATTGTGCACGTTG
PKCβII	GACCAAACACCCAGGCAAAC	GATGGCGGGTGAAAAATCGG
INF2	CACATCCAACGTGATGGTGAAG	GGAGAGCTCGTTCATGACAATG

## Supplementary Materials

The following are available online at https://www.mdpi.com/2072-6694/12/9/2391/s1, Figure S1: YAP/TAZ target genes’ induction in B16-F0 murine cell line, Figure S2: Ultra low attachment conditions, Figure S3: Correlation analysis between TPCN2 and Vimentin; MITF; ZEB1; CTGF; ANKRD1; CYR61; CHD-2(N-Cadherin); MMP9 in primary patients in human skin melanoma TCGA database, Figure S4: Correlation analysis between TPCN2 and Vimentin; MITF; ZEB1; CTGF; ANKRD1; CYR61; CHD-2(N-Cadherin); MMP9 in metastatic patients in human skin melanoma TCGA database, Table S1: Statistical analysis of TPC2 and other genes’ correlation in skin melanoma TGCA dataset.
