# Supplementary material for: Loss of Two-Pore Channel 2 (TPC2) Expression Increases the Metastatic Traits of Melanoma Cells by a Mechanism Involving the Hippo Signalling Pathway and Store-Operated Calcium Entry
Source: Cancers (Basel). 2020 Aug 24;12(9):2391. doi: 10.3390/cancers12092391 (PMC7564716; doi:10.3390/cancers12092391)
Supplement: Supplementary file 1 [file cancers-12-02391-s001.pdf]

cancers

MDPI

Loss of Two-Pore Channel 2 (TPC2) Expression Increases the Metastatic Traits of Melanoma Cells by a Mechanism Involving the Hippo Signalling Pathway and Store-Operated Calcium Entry

Antonella D'Amore, Ali Hanbashi, Silvia Di Agostino, Fioretta Palombi, Andrea Sacconi, Aniruddha Voruganti, Marilena Taggi, Rita Canipari, Giovanni Blandino, John Parrington and Antonio Filippini

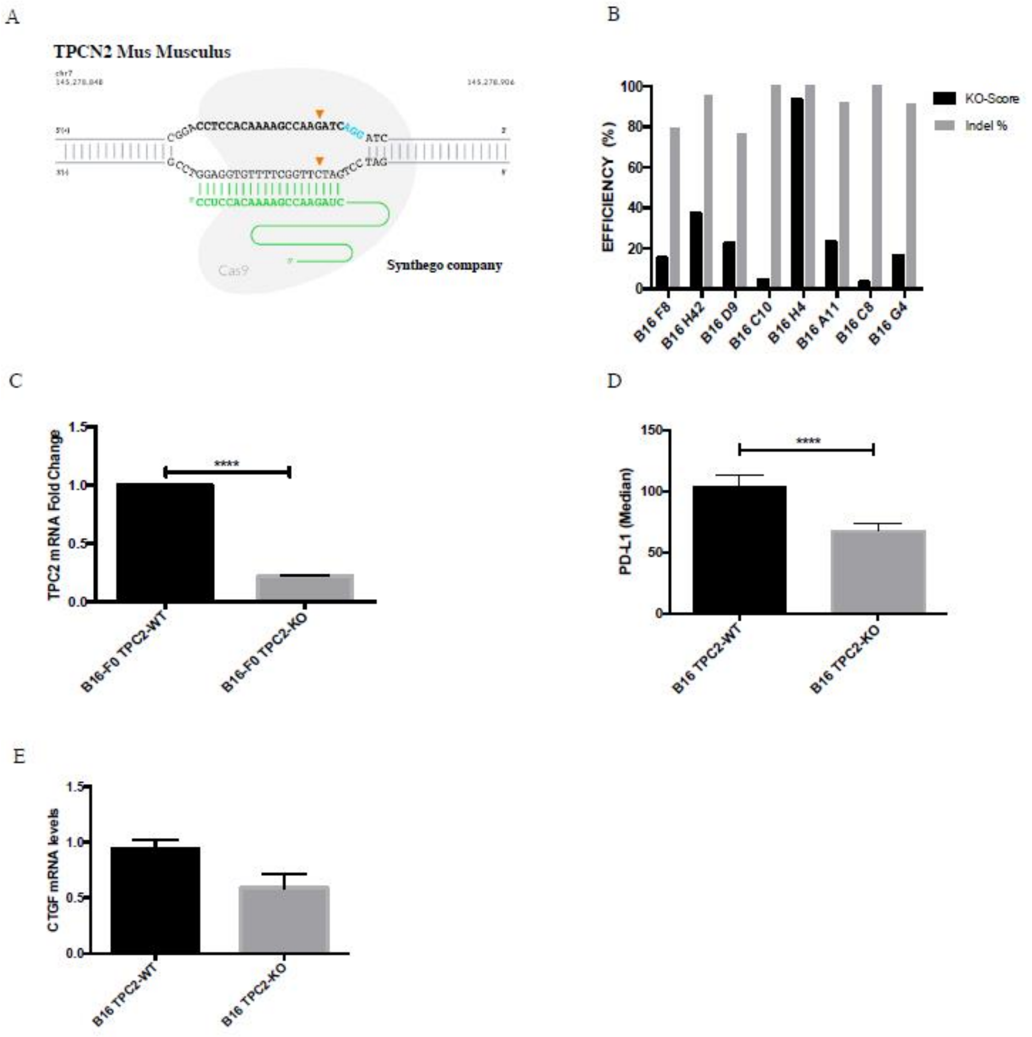

Figure S1. YAP/TAZ target genes' induction in the B16-F0 murine cell line. (**A**) Schematic of CRISPR-Cas9 strategy used to target the B16-F0 murine melanoma cell line. (**B**) ICE Analysis of possible KO clones, from which the B16 clone H4 was selected. KO-Score indicates the percentage sequences that are putative knockout. (**C**) qPCR analysis of TPC2 transcript levels in B16 -F0 (WT and KO) cells. (**D**) Flow cytometric detection of PD-L1 in B16 WT and TPC2 KO cells. (**E**) q-PCR analysis of YAP/TAZ target genes in B16 WT and TPC2 KO cells. GAPDH was used as an internal control. Data in bar charts represents mean ± s.e.m. of three independent experiments. (**** $p < 0.0001$)

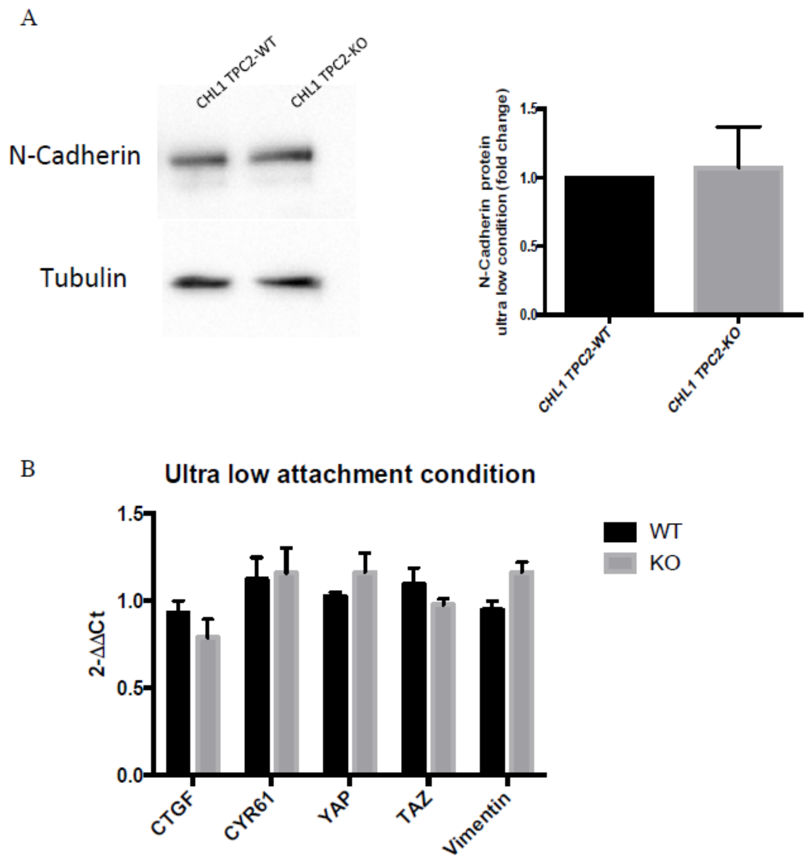

Figure S2. Ultra low attachment conditions. (**A**) N-cadherin protein expression in CHL1 WT and TPC2 KO cells, normalised to tubulin. Relative density of N-cadherin/tubulin. (**B**) qPCR analysis of YAP/TAZ target genes in CHL1 WT and TPC2 KO cells. H3 was used as an internal control. ANKRD1 is no longer expressed in the ultralow attachment condition but is expressed only in the attached condition. Data in bar charts represents mean ± s.e.m. of three independent experiments.

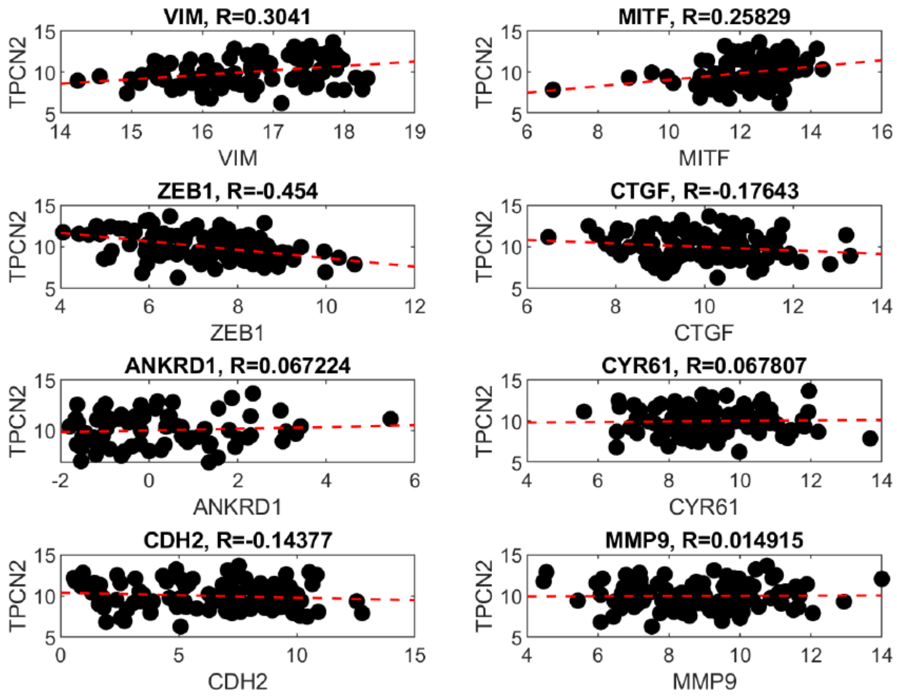

Figure S3. Correlation analysis between TPCN2 and Vimentin; MITF; ZEB1; CTGF; ANKRD1; CYR61; CHD-2 (N-Cadherin); MMP9 in primary patients in human skin melanoma TCGA database.

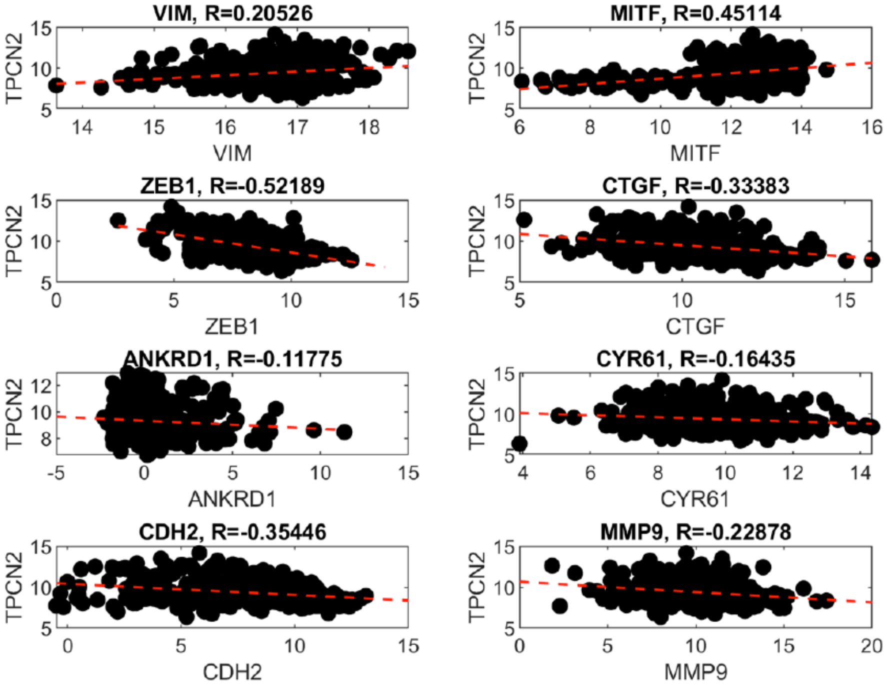

Figure S4. Correlation analysis between TPCN2 and Vimentin; MITF; ZEB1; CTGF; ANKRD1; CYR61; CHD-2 (N-Cadherin); MMP9 in metastatic patients in human skin melanoma TCGA database.

Table S1. Statistical analysis of TPC2 and other genes' correlation in skin melanoma TGCA dataset.

**All Patients (n = 471)**				
**Gene**	**Spearman'R**	***p***	**Pearson'R**	***p***
TPCN2\VIM	0.229099414	5.39523×10^{-7}	0.263582515	6.31354×10^{-9}
TPCN2\MITF	0.409046745	0	0.376648898	2.53303×10^{-17}
TPCN2\ZEB1	−0.5297336	0	−0.534623769	3.57548×10^{-36}
TPCN2\CTGF	−0.319158928	1.71671×10^{-12}	−0.311525576	4.65348×10^{-12}
TPCN2\ANKRD1	−0.096831987	0.063873392	−0.093199051	0.074548989
TPCN2\CYR61	−0.144347794	0.001700518	−0.133714264	0.00364571
TPCN2\CDH2	−0.317477969	1.82068×10^{-12}	−0.272286733	1.96018×10^{-9}
TPCN2\MMP9	−0.213695416	3.03597×10^{-6}	−0.207476406	5.61751×10^{-6}
**Primary Patients (n = 103)**				
**Gene**	**Spearman'R**	***p***	**Pearson'R**	***p***
TPCN2\VIM	0.304100953	0.001868945	0.304018882	0.001795976
TPCN2\MITF	0.258291965	0.008591619	0.271398533	0.00555374
TPCN2\ZEB1	−0.454004305	1.92035×10^{-6}	−0.437800374	3.75321×10^{-6}
TPCN2\CTGF	−0.176426657	0.074688402	−0.172551613	0.081345121
TPCN2\ANKRD1	0.067223513	0.574754805	0.081098765	0.498266976
TPCN2\CYR61	0.067807407	0.495538832	0.029742943	0.765520035
TPCN2\CDH2	−0.143774986	0.147179955	−0.117116415	0.238734705
TPCN2\MMP9	0.014914554	0.880971271	0.012218954	0.902503073
**Metastatic Patients (n = 368)**				
**Gene**	**Spearman'R**	***p***	**Pearson'R**	***p***
TPCN2\VIM	0.205256862	7.55181×10^{-5}	0.24300457	2.39745×10^{-6}
TPCN2\MITF	0.451140773	0	0.3996398	1.52261×10^{-15}
TPCN2\ZEB1	−0.521890588	0	−0.541720691	1.88456×10^{-29}
TPCN2\CTGF	−0.333832253	6.51323×10^{-11}	−0.328853262	9.94384×10^{-11}
TPCN2\ANKRD1	−0.117753414	0.043286936	−0.104126915	0.074147415
TPCN2\CYR61	−0.164351698	0.001577769	−0.150544712	0.003795965
TPCN2\CDH2	−0.354464985	2.6365×10^{-12}	−0.297842918	5.92101×10^{-9}

TPCN2\MMP9	−0.228782183	9.83235×10^{-6}	−0.220852783	1.90981×10^{-5}

© 2020 by the authors. Licensee MDPI, Basel, Switzerland. This article is an open access article distributed under the terms and conditions of the Creative Commons Attribution (CC BY) license (http://creativecommons.org/licenses/by/4.0/).
